# Heatmap-Guided Selective Feature Attention for Robust Cascaded Face Alignment

**DOI:** 10.3390/s23104731

**Published:** 2023-05-13

**Authors:** Jaehyun So, Youngjoon Han

**Affiliations:** 1 Department of Electronic Engineering, Soongsil University, Seoul 06978, Republic of Korea; ru2ror@gmail.com; 2 School of AI Convergence, Soongsil University, Seoul 06978, Republic of Korea

**Keywords:** face alignment, feature attention, heatmap regression, coordinate regression, multi-task learning

## Abstract

Face alignment methods have been actively studied using coordinate and heatmap regression tasks. Although these regression tasks have the same objective for facial landmark detection, each task requires different valid feature maps. Therefore, it is not easy to simultaneously train two kinds of tasks with a multi-task learning network structure. Some studies have proposed multi-task learning networks with two kinds of tasks, but they do not suggest an efficient network that can train them simultaneously because of the shared noisy feature maps. In this paper, we propose a heatmap-guided selective feature attention for robust cascaded face alignment based on multi-task learning, which improves the performance of face alignment by efficiently training coordinate regression and heatmap regression. The proposed network improves the performance of face alignment by selecting valid feature maps for heatmap and coordinate regression and using the background propagation connection for tasks. This study also uses a refinement strategy that detects global landmarks through a heatmap regression task and then localizes landmarks through cascaded coordinate regression tasks. To evaluate the proposed network, we tested it on the 300W, AFLW, COFW, and WFLW datasets and obtained results that outperformed other state-of-the-art networks.

## 1. Introduction

The human face provides crucial information for understanding user behavior in human–computer interactions and has been studied in computer vision for a long time. Many methods for analyzing face attributes, such as facial expression recognition [1,2] and head pose estimation [3,4], detect facial regions during the preprocessing step. The face region detection method is divided into the face bounding box, which defines the position of the rectangular region of the face, and face alignment, which extracts the optimal face region. Face alignment is a method for detecting facial landmarks, which are key points representing facial components. It improves the performance of applications by extracting information, such as a face component, size, rotation, and position. Although deep learning algorithms have improved face alignment capabilities in recent years, there is still a need to improve performance in noisy environments.

Face alignment studies using deep neural networks have focused on coordinate and heatmap regression. Coordinate regression directly estimates facial landmark coordinates and is designed based on common deep neural network structures, such as VGG [5] and ResNet [6]. Heatmap regression in face alignment has been studied since Newell et al. [7] first proposed a stacked hourglass network. Heatmap regression methods exhibit robust performance against noise by estimating the probability that a landmark exists at a pixel location. However, the accuracy of landmark locations largely depends on the resolution of the heatmap because pixel units are expressed as integers. This issue is known as the quantization error problem [8].

Recently, hybrid methods with coordinate and heatmap regression tasks have been proposed [9,10]. Wu et al. [9] trained on these tasks independently and sequentially. Park et al. [10] trained coordinate and heatmap regression tasks simultaneously, but their method did not perform better than the sequential training method.

Although these regression tasks have the same objective for facial landmark detection, each task requires different valid feature maps. Therefore, it is not easy to simultaneously train two kinds of tasks with a multi-task learning network structure. Some studies have proposed multi-task learning networks with two kinds of tasks, but they still need to propose an efficient network that can train them simultaneously due to the shared noisy feature maps.

We propose heatmap-guided selective feature attention for robust cascaded face alignment based on multi-task learning, which improves the performance of face alignment by efficiently training heatmap and coordinate regression tasks. The attention module can select valid feature maps without losing the properties of low- and high-level feature maps, and the cascaded coordinate regression network improves the performance of face alignment using the backward propagation connection for tasks. This study also employs a refinement strategy that detects global landmarks through a heatmap regression task and then localizes landmarks through cascaded coordinate regression tasks.

**Figure 1:**
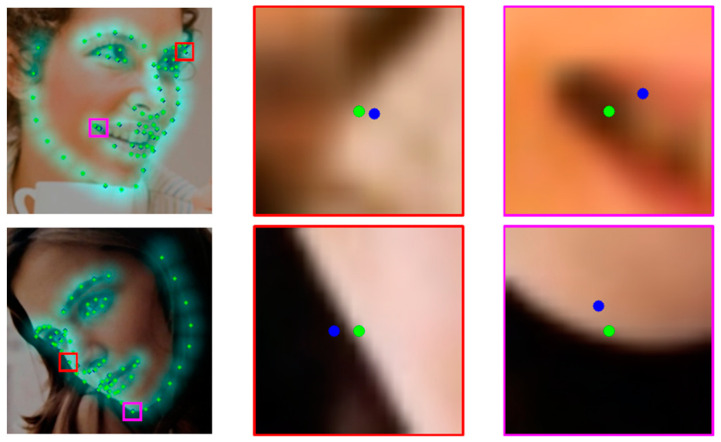
The first column shows the predicted landmarks, and cyan regions express landmark heatmaps. The regions in the red and magenta boxes are expressed in the second and third columns. The blue dot is the maximum probability location of the heatmap, and coordinate regression networks predict the green dot. The second and third columns show a close-up of the bounding box around the landmark in the first column image.

Heatmap regression estimates a heatmap using stacked hourglass networks and converts it to landmark coordinates. The estimated heatmap and converted landmark coordinates are used as information in the cascaded coordinate regression (CCR) stage. CCR detects local facial landmarks using region of interest (ROI) feature maps around the landmark coordinates in the previous CCR stage. The result of each CCR stage is propagated as input information for the next CCR stage. Figure 1 shows examples of accurate searches using a CCR network.

To verify the effectiveness of the network, we evaluated popular face alignment benchmarks, including 300W [11], AFLW [12], COFW [13], and WFLW [9]. This study compared the performance of the proposed network with that of previous state-of-the-art networks. Our main contributions are summarized as follows:

1. We propose an effective attention method by selecting multi-level features through the estimated heatmap.
2. We propose backward propagation connections between a heatmap regression network and a coordinate regression network for effective multi-task learning to improve performance.
3. We designed a heatmap-cascaded coordinate regression network and verified its performance for the proposed network through ablation studies.

## 2. Related Work

Traditional face alignment methods include the active appearance model [14] and the constrained local model [15], based on a dimension-reduction technique using principal component analysis. These methods can express facial features in low dimensions and work well indoors. A method using shape-indexed features [16,17] has demonstrated the possibility of face alignment in the wild. In recent years, deep-learning-based coordinate and heatmap regression have been proposed and have shown good performance in noisy environments.

### 2.1. Coordinate Regression Methods

Coordinate regression directly estimates the position of facial landmarks. These methods have improved performance with the development of backbone networks, such as VGG [5] and ResNet [6], and additional methods for face alignment. Feng et al. [18] proposed a Wing loss to increase the training contribution of samples with small loss values. Su et al. [19] initialized facial landmarks using a ResNet-based network and searched for regions around the location found in a previous network. Li et al. [20] and Lin et al. [21] demonstrated robust performance in the presence of occlusions using a graph convolutional layer. Xu et al. [22] addressed large poses using multiple predefined landmark templates. Zheng et al. [23] proposed a pre-training method based on contrastive learning using extra datasets. Li et al. [24] and Xia et al. [25] proposed a transformer structure, but the transformer module estimated displacements of facial landmarks using local patches. These coordinate regression methods have been studied for their fast processing and accurate performance.

### 2.2. Heatmap Regression Methods

Heatmap regression indirectly estimates the positions of facial landmarks using a heatmap. A heatmap expresses the probabilities of landmark existence, and high probabilities can be regarded as candidates for facial landmarks. Since the stacked hourglass network [7] was proposed for human pose estimation, heatmap regression for face alignment has also employed an encoder–decoder structure. Bulat et al. [26] and Yang et al. [27] initially proposed the same network as the stacked hourglass network [7]. Wang et al. [28] proposed an AWing loss to reduce the problem caused by many background pixels in a heatmap. Zhang et al. [29] designed an hourglass network using an Inception-Resnet module [30] and refined the landmark coordinates estimated by the heatmaps. Huang et al. [31] proposed an attention module that converts a landmark heatmap into a boundary heatmap. Lan et al. [8] expressed an offset from a previously estimated landmark position to the ground truth as a local high-resolution heatmap to solve the quantization error caused by a lower-resolution heatmap. Jin et al. [32] estimated not only the landmark heatmap but also an offset heatmap and a neighbor heatmap, which indicate the distance to a neighboring landmark. Bulat et al. [33] proposed a Siamese-based training method. Heatmap regression methods have shown good results on various benchmarks, and many methods have recently been proposed for solving quantization errors.

### 2.3. Hybrid Methods

The hybrid model consists of a heatmap and a coordinate regression task. Valle et al. [34,35] initialized landmarks using a heatmap regression and refined the initialized landmarks using ensemble regression trees [17], which is a traditional method of shape-indexed features. Wu et al. [9] estimated a boundary heatmap and applied it to a ResNet-based coordinate regression task. Park et al. [10] improved the performance by converting the results of the coordinate regression task into a heatmap and combining them with the results of the heatmap regression task. In these methods, the heatmap and coordinate regression tasks were not trained simultaneously but were trained separately. It has a limited effect on multi-task learning [36] because the information needs to be propagated interactively during the training of each task.

### 2.4. Multi-Task Learning

Multi-task learning in the face alignment field has mainly been studied to estimate other facial attributes along with landmark detection. Ranjan et al. [37] proposed a network that estimates gender and pose, together with facial landmark detection. Kumar et al. [38] estimated the location, probability distribution, and visibility of landmarks. Prados et al. [39] initialized landmarks using a head pose and estimated landmark displacements. Although multi-task learning in the face alignment field efficiently estimates various facial attributes, it does not describe how facial attribute estimations improve face alignment performance except through landmark initialization methods.

## 3. Proposed Network

**Figure 2:**
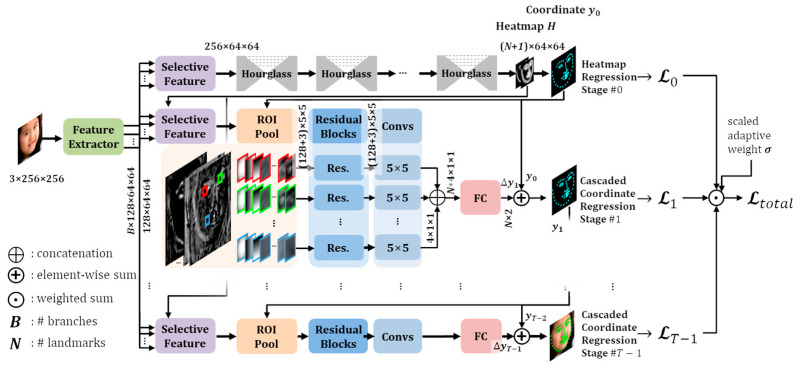
Overview of the proposed network structure. Each task shares layer weights in the feature extraction. In the first stage, heatmaps are regressed by stacked hourglass networks. The predicted result is then propagated to the next stage and used to regress the offset coordinates.

The structure of the proposed face alignment framework is illustrated in Figure 2. The proposed network consists of a feature extractor, selective feature modules for each task, a heatmap regression network for global landmark detection, and a CCR network for local landmark detection. The proposed network has a refinement structure based on multi-task learning, in which the heatmap regression and CCR tasks can be trained simultaneously, and the information from each task is transferred to other tasks. We added attention modules for selecting valid feature maps and designed backward propagation for effective multi-task learning.

### 3.1. Heatmap-Guided Selective Feature Attention

Low-level feature maps in deep neural networks have a lot of noise and unrefined information and are mainly composed of spatially filtered information, such as edges [19]. By contrast, high-level feature maps have less noise as the layer deepens and contain important semantic information for the output. As the feature maps of each level are sequentially filtered toward the target, the rich information in low-level feature maps is gradually reduced. The effectiveness of low-level feature maps has been demonstrated using fully convolutional networks [40] and feature pyramid networks [41]. In the face alignment field, Lin et al. [21] also demonstrated the effectiveness of multi-level features using convolutional block attention modules (CBAM) [42] for removing noisy information from low-level feature maps.

**Figure 3:**
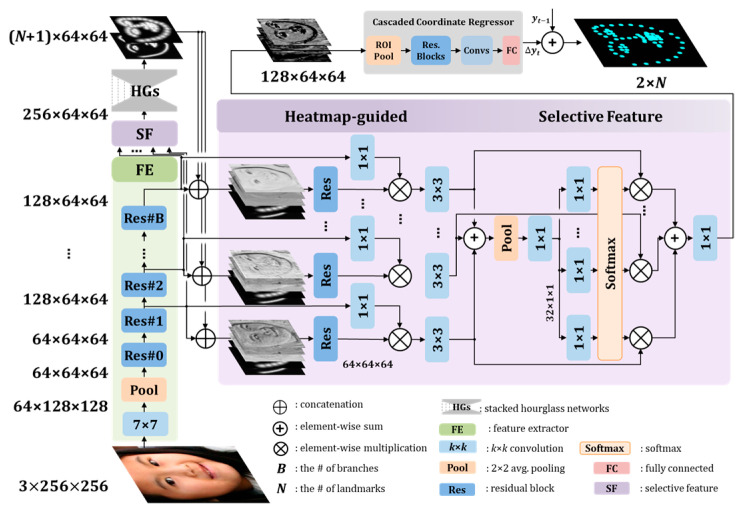
Structure of the feature extractor and heatmap-guided selective feature module.

We propose selective feature attention (Figure 3) that selects multi-level feature maps. The attention module can select valid feature maps without losing the properties of multi-level feature maps. We used the estimated heatmap to improve the CCR performance before attention. They are filtered using convolution layers as

(1) $$U_{i,t}=\{\begin{array}{ccc} F_{3\times 3,i,t}(F_{1\times 1,i,t}^{0}(F_{i})) & , & \mathrm{if}\;t=0\\ F_{3\times 3,i,t}(F_{1\times 1,i,t}^{0}(F_{i})⊗F_{res,i,t}(F_{i}⊕H)) & , & \mathrm{otherwise} \end{array}$$

where i is an index of the branch, t is an index of the task stage, ⊕ is the concatenation, F is the feature map, H is the heatmap, $F_{res}$ is the residual block, and $F_{1\times 1}$ and $F_{3\times 3}$ are the convolution operations. The superscript on $F_{1\times 1}$ is the order of the network layers. The filtered feature maps of each branch are combined using

(2) $$z_{t}=(F_{1\times 1,t}^{1}○F_{gap})(\sum_{i=0}^{B-1}U_{i,t})$$

In Equation (2), the feature maps U are integrated through an element-wise summation and calculated using the global average pooling $F_{gap}$ and convolution layer $F_{1\times 1,t}^{1}$. The value of z is then calculated using $F_{1\times 1,i,t}^{2}$ for each branch excitation, and the final scale $s_{i,t}$ is calculated using the softmax function as

(3) $$s_{i,t}=\frac{e^{F_{1\times 1,i,t}^{2}(z_{t})}}{\sum_{j=1}^{B}e^{F_{1\times 1,j,t}^{2}(z_{t})}}$$

The final feature map $F_{t}^{att}$ is calculated through multiplication of $s_{i,t}$ and $U_{i,t}$.

(4) $$F_{t}^{att}=F_{1\times 1,i,t}^{3}(\sum_{i=1}^{B}s_{i,t}U_{i,t})$$

**Figure 4:**
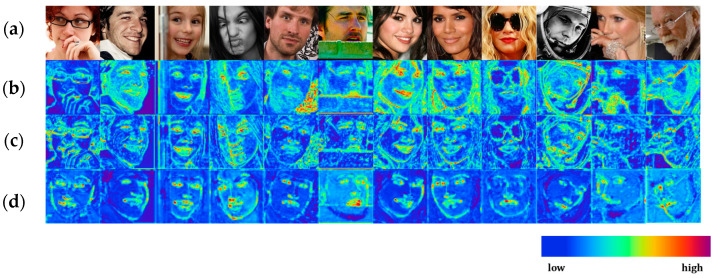
Effectiveness of the selective feature module. The illustrations are expressed by the average feature maps before the CCR network. The brighter the color in the feature map, the higher the feature value. The feature maps are focused on facial parts by the selective feature module. (**a**) Input images; (**b**) feature maps without the selective feature module; (**c**) feature maps after the selective feature module without the heatmap; (**d**) the feature maps after the heatmap-guided selective feature.

The dimensions of the feature maps were adjusted using the output of a 1 × 1 convolution layer. The effectiveness of the selective feature module is shown in Figure 4. The feature maps were brightly expressed on the face. In addition, we also verified that the heatmap causes the feature map to focus on the facial components and reduce noise.

### 3.2. Designing Backward Propagation Connections

In the proposed network, as shown in Figure 2, the selective feature module and summation component are essential for exchanging information between stage tasks. The selective feature module connects the feature extractor to the network layer of all stage tasks, and the summation component connects the network layer of the previous stage task to the network layer of the current stage task. Therefore, the learning result for each stage task in the proposed network is significantly affected by the backward propagation connection structure of the summation component.

**Figure 5:**
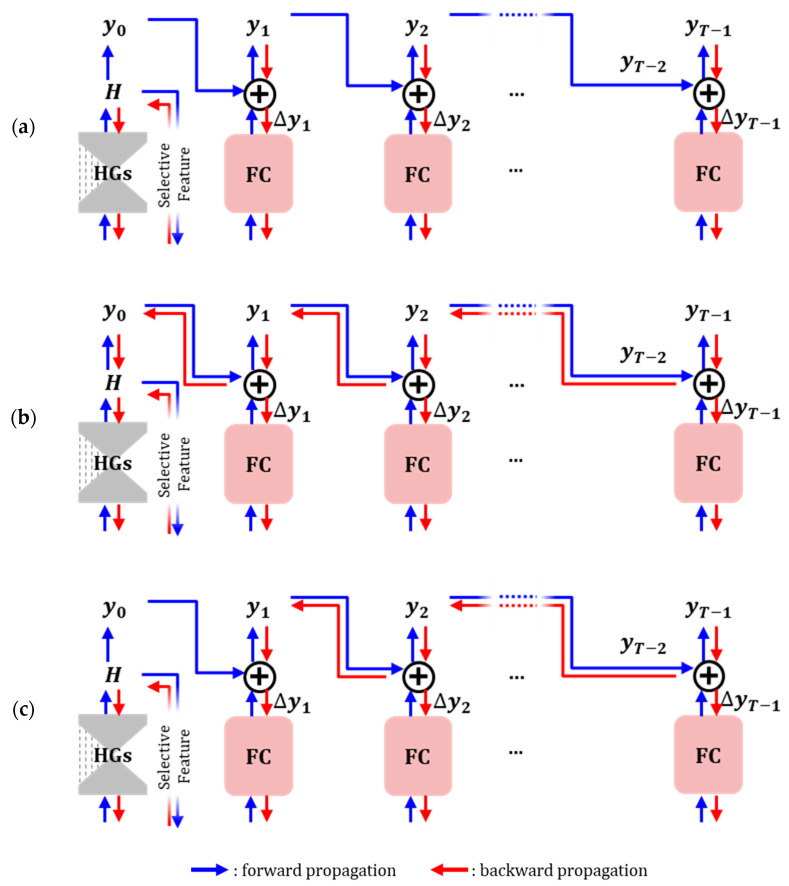
Designed backward propagations. Blue arrows denote the forward pass, and red arrows denote the backward pass. (**a**) Task-wise connection design shares the weights of only the feature extractor. (**b**) In the fully connected design, information from a CCR offset backward propagates to the heatmap regression network. (**c**) In a CCR connection, information from the first CCR does not propagate backward to the heatmap regression network.

We designed backward propagation connections for the summation component to control the learning influence between the network layers of each stage task. Figure 5 shows the three backward propagation connections between the network layers of the tasks.

- Task-wise connection: No backward propagation connection for the summation component in all stage tasks (Figure 5a). It is a common structure for multi-task learning, and all tasks share the feature extractor module in the early network layer. The shared feature extractor prevents overfitting for a single task type. Because the feature extractor module is a front-end network module, it slightly impacts performance.
- Fully connection: A backward propagation connection for the summation component in all stage tasks (Figure 5b). The backward information of tasks affects not only the feature extractor shared by all tasks but also task-specific layers. Because the information from the neighbor stage is backward-propagated to the specific task layers, an improvement or deterioration of performance is clearly observed for the backward propagation of the neighbor stage.
- CCR connection: Having a backward propagation connection for the summation component only in the CCR tasks, except in the heatmap regression task (Figure 5c). Compared with the full connection, it removes the backward propagation connection between the heatmap regression task and the first CCR task. By not propagating the bad backward information of the first CCR task to the heatmap regression network, it improves the performance of CCR tasks and makes the training for each task manageable.

We used the CCR connection in the proposed network and evaluated the performance of the types in the ablation study described in Section 5.3.

### 3.3. Cascaded Face Alignment Network with Heatmap-Guided Selective Feature

The cascaded face alignment network with heatmap-guided selective features (CHS) is the proposed network structure in this study. The heatmap regression task estimates the probability that a landmark exists in each pixel, and the coordinate regression task predicts the position of the landmark in the image. Although they have the same objective for facial landmark detection, each task has parameters with different units and scales. Therefore, it is not easy to simultaneously train two kinds of tasks with a multi-task learning network structure. For effective multi-task learning, the proposed network is composed of four types of modules, as shown in Figure 2.

- Feature extractor: The feature extractor extracts feature maps from an input image, and they are shared by all tasks. It consists of a convolution layer and *B* + 1 residual blocks for *B* input branches of the selective feature module.
- Selective feature module: The selective feature module in this paper selects valid feature maps from several branches extracted from the feature extractor.
- Heatmap regression network: The heatmap regression network estimates landmark heatmaps and a boundary heatmap, such as the stacked hourglass network in AWing [28].
- Cascaded coordinate regression network: The CCR network extracts the ROI feature map for each landmark through the ROI pooling layer and concatenates the coordinate channels [43]. The coordinate channels that represent the coordinates in the feature map can improve coordinate regression performance by concatenating the original feature channels [28,43]. In this paper, the coordinate channels are concatenated to the feature map for each landmark to improve CCR performance. Here, the ROI feature map was independently created through a residual block and a convolution layer for each landmark. The feature maps were concatenated in the last layer and used to estimate the offset coordinates using the fully connected layer. The global landmark coordinates of the current stage were obtained by adding the estimated offset to the global landmark coordinates of the previous stage.

**Figure 6:**
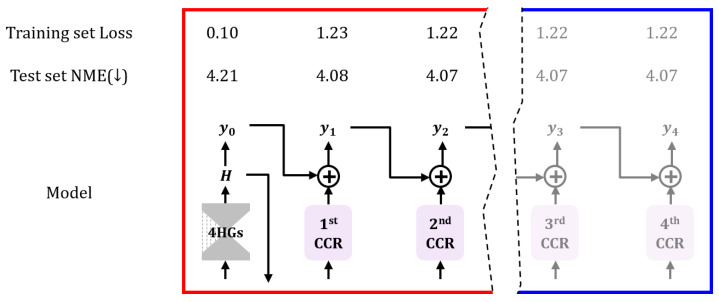
Pruned CCR networks by checking the training set loss and test set NME at each stage. Down arrow in the figure means a propagation of an estimated heatmap. We obtained an experimental result of the efficient CCR for the performance. The red box is the efficient network set, and the blue box is the inefficient set.

We experimentally found that the CHS with the 4-CCRs has the best performance (Section 5.1), but there is only a slight difference in performance at each stage of the CCR network. The network parameters can be reduced by pruning the stage CCR network after training. Figure 6 shows the loss value and NME measured at each CCR stage of the last epoch of the trained heatmap-4CCRs model for the 300W dataset. This study experimentally confirmed an effective performance improvement for the second CCR of four CCRs. Finally, we pruned the third and fourth CCR networks from the entire model after the training.

**Figure 7:**
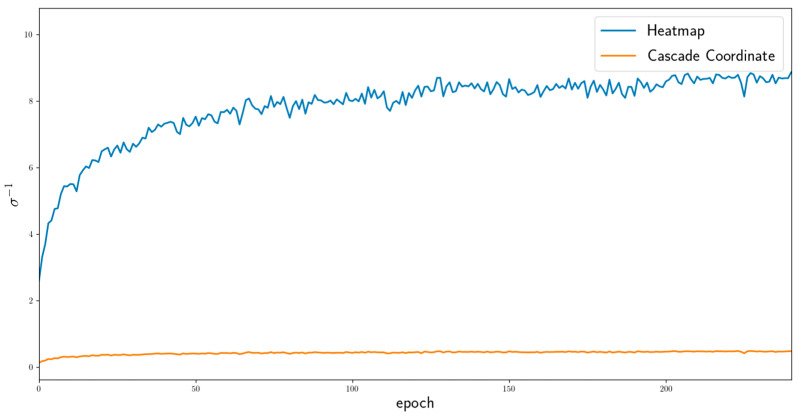
Loss weights in the training step from 0 to 240 epochs. The blue line shows the heatmap loss weight, and the orange line indicates the first coordinate loss weight.

The final loss function in multi-task learning is a combination of task losses. To overcome training problems caused by different units and scales of task losses, it was used as a weighted sum of task losses using fixed weights in previous studies [37,38], which dealt with face alignment and other facial attribute classifications. However, the performance of this approach largely depends on predefined weights. Kendall et al. [44] proposed adaptive weights through maximum likelihood inference, as shown in Equation (5), assuming that each task follows a Gaussian distribution to determine the optimal weights for image segmentation and depth regression loss.

(5) $$L_{\mathrm{adaptiveWeight}}=\sum_{t=0}^{T-1}\left(\frac{1}{e^{\ln\sigma_{t}}}L_{t}+\ln\sigma_{t}\right)$$

where L is the loss, T is the number of tasks, and σ is a trainable parameter adjusted to suit the training state. Figure 7 shows the change in the adaptive weight proposed in this paper. The weight of the heatmap regression task loss is significantly larger than that of the coordinate regression task loss, and the difference between them increases as training progresses. The significant difference makes it difficult to determine the optimal weights. To reduce the difference, we first used the fixed loss weights and then alleviated them using the adaptive loss weights.

(6) $$L_{\mathrm{scaledAdaWeight}}=\sum_{t=0}^{T-1}\alpha_{t}(\frac{1}{e^{\ln\sigma_{t}}}L_{t}+\ln\sigma_{t})$$

In Equation (6), α is a fixed weight. The proposed method uses the AWing loss [28] for heatmap regression, and the log function in the Wing loss [18] was used for coordinate regression (Equation (7)).

(7) $${\mathrm{Wing}}_{small}(∆y,∆\hat{y})=w_{\mathrm{wing}}\ln\left(1+\left|∆y-∆\hat{y}\right|/\epsilon_{\mathrm{Wing}}\right),∆\hat{y}=y-\hat{y}$$

∆y is the estimated offset, and $∆\hat{y}$ is the ground-truth offset. We did not use a linear function of the Wing loss because the ROI constrains coordinate regression. The large error caused by the offset estimated outside the ROI makes it difficult to reach the global minimum of the loss function during the training. In this study, because a large error is calculated as a small error using the log function, it has little effect on the shared layer. The hyperparameters of the heatmap loss function and the CCR loss function are the same as those in the AWing [28], and the Wing loss was set to $w_{\mathrm{wing}}=8.0$ and $\epsilon_{\mathrm{Wing}}=10.0$. The final loss function is shown in Equation (8):

(8) $$L_{\mathrm{total}}=\alpha_{0}(\frac{1}{e^{\ln\sigma_{0}}}\mathrm{AWing}(H,\hat{H})+\ln\sigma_{0})+\sum_{t=1}^{T-1}\alpha_{i}(\frac{1}{e^{\ln\sigma_{t}}}{\mathrm{Wing}}_{small}(∆y,∆\hat{y})+\ln\sigma_{t})$$

H is the estimated heatmap and $\hat{H}$ is the ground truth heatmap.

## 4. Experiments

To demonstrate the effectiveness of the proposed method, we conducted experiments on four popular benchmark datasets: 300W [11], AFLW [12], COFW [13], and WFLW [9]. The 300W dataset is the most widely used, and the 300W private test dataset is used for the competition of models trained on the 300W public dataset. AFLW focuses on large poses, whereas COFW focuses on large occlusions. WFLW is currently the most challenging dataset and can be used to evaluate the performance of each noise by providing attributes. Since the proposed method does not address a training method using extra data, we compared the proposed method to state-of-the-art training from scratch. Results of state-of-the-art studies [9,23,24,33] known to have used extra data were excluded for a fair comparison.

### 4.1. Evaluation Metrics

#### 4.1.1. Normalized Mean Error

The normalized mean error (NME) was used to evaluate the distance between the facial landmark detection result and ground truth as

(9) $$NME=\frac{1}{N}\sum_{i=0}^{N-1}\frac{{\left\|x-\hat{x}\right\|}_{2}^{2}}{d}$$

where $\hat{x}$ is the predicted landmark, x is the ground truth, *N* is the number of landmarks, and *d* is the normalization factor. We employed the inter-pupil distance (IPD) and inter-ocular distance (IOD) as normalization factors on the 300W and COFW datasets. The IPD is the distance between the centers of the two eyes, and the IOD is the distance between the outer endpoints of the two eyes. AFLW uses the face size and 300W private test dataset, and WFLW uses the IOD.

#### 4.1.2. Failure Rate

The failure rate (FR) is another metric for evaluating the quality of the detection performance and indicates the ratio of samples for which the NME exceeds the threshold in all samples. It can be interpreted that the larger the FR value, the more failed samples there are. In this study, to evaluate the COFW and WFLW datasets, the threshold of the NME was defined as 10%

#### 4.1.3. Area under the Curve

The area under the curve (AUC) is calculated by integrating the cumulative error distribution (CED) curve. The CED can be expressed as a curve by connecting the ratio of the samples to the corresponding NME. In general, the curves are expressed with an NME below a certain threshold, which we define as 7% for the AFLW dataset and 10% for the COFW and WFLW datasets.

### 4.2. Implementation Details

We cropped only face images for all the training and test sets using the bounding box provided by the dataset. Because no bounding box was provided for the 300W private test dataset, we used a ground truth bounding box created by the outermost landmarks of the ground-truth landmark. The cropped facial images were resized and used as inputs to the network.

In the heatmap regression network, the heatmap size was 64 × 64 pixels. We used a 4-stacked hourglass network. The heatmap regression network estimates landmark heatmaps and boundary heatmaps [28]. The input feature maps of the selective feature had dimensions of 64 × 64 × 128 pixels and used three branches. ROI pooling in the coordinate regression network applied a fixed ROI with a pixel resolution of 5 × 5 pixels. The residual block in the heatmap regression network used a hierarchical, parallel, and MS block [45], whereas the residual block in the coordinate regression network used a basic block [6]. We set the fixed loss weights to 1.0, except for the last CCR loss weight of 3.0.

Data augmentation was initially applied with ±15% random scaling, random rotation of ±50°, random translation of ±25 pixels, 50% flipping, and 0–50% occlusion [10]. A transform, such as random Gaussian noise, grayscale, contrast, color, power-law transform, histogram equalization, JPEG compression artifacts, lighting, or identity, was then randomly selected and applied.

The coordinate transformation in the heatmap was conducted in the same manner as that used by Newell et al. [7]. We trained the network model using the SGD optimizer and set the momentum to 0.9 and the weight decay to 5 × 10^−4^. The batch size was 10, and the learning rate schedule was as follows:

(10) $$lr_{k}=\{\begin{array}{lll} 0.75\times lr_{k-1} & , & \mathrm{if}\;k\;\mathrm{in}\;a\\ lr_{k-1} & , & \mathrm{otherwise} \end{array}\;a=\{120,200,240,245,250,255,\ldots ,330\}$$

where k is the epoch, and the initial learning rate $lr_{0}$ is 1 × 10^−2^. This learning rate schedule was experimentally determined, and networks are trained from scratch using only the training set provided by datasets. We applied the same data augmentation, learning schedule, and network structure to all datasets. The only difference in the implementation is the fully connected layer at the final output of the network due to the different number of landmarks in each dataset.

### 4.3. Evaluation

#### 4.3.1. Evaluation of 300W

**Figure 8:**
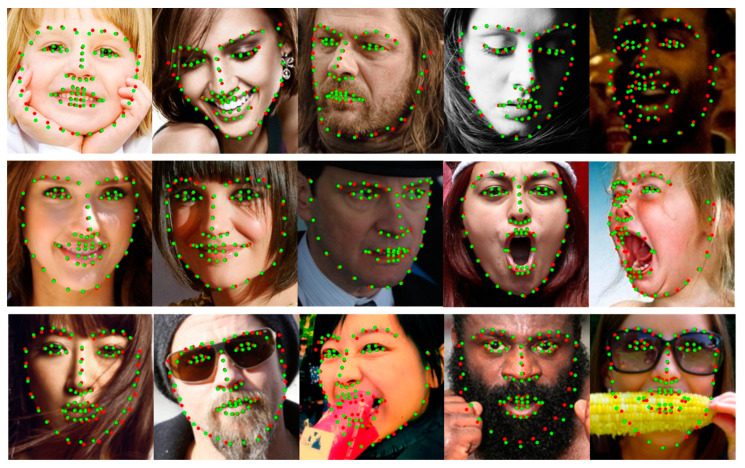
Visualized examples on the 300W dataset. Green dots denote predictions by CHS, and red dots denote the ground truth. The first row is the 300W public test set, the second row is the 300W private test dataset, and the third row is the COFW-68 dataset.

The 300W dataset consists of 3148 images as a training set and 689 as a test set with 68 landmarks. The test set was divided into a common subset with less noise and a challenging subset with large poses and occlusions. The 300W private test dataset consists of 300 images as an indoor subset and 300 as an outdoor subset. COFW-68 [46] provides 68 landmarks for the test set of COFW. The CHS trained on the 300W dataset is also evaluated on the 300W private test and the COFW-68 dataset. Figure 8 shows the results of the CHS for large occlusions. As shown in Table 1, the CHS showed performance improvements of 0.3%, 2.2%, and 0.7% in the common subset, challenging subset, and fullset, respectively, compared to ADNet [47] in the IOD evaluation. The CHS also showed performance improvements of more than 2.3% compared to SPIGA [39] on the 300W private test set and 1.3% compared to ACN [10] on the COFW-68 dataset, as shown in Table 2.

#### 4.3.2. Evaluation of AFLW

**Figure 9:**
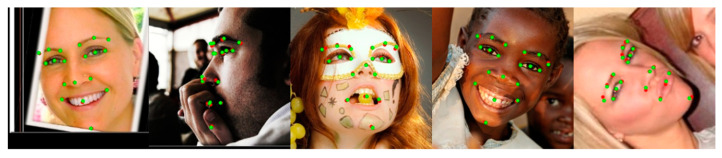
Visualized examples on the AFLW dataset. Green dots denote predictions by CHS, and red dots denote the ground truth.

**Figure 10:**
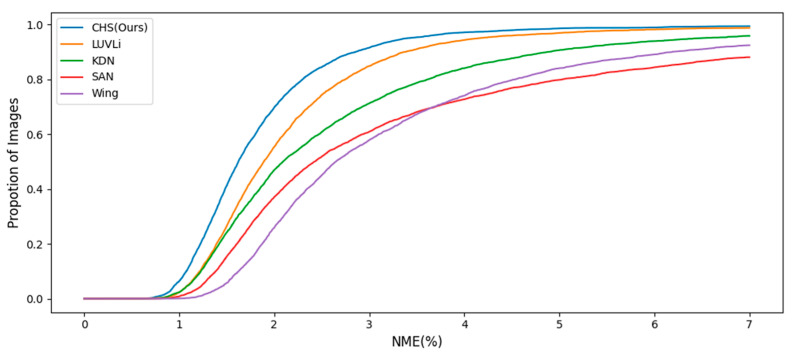
CED curves of different methods using the ground truth bounding box on AFLW.

AFLW consists of 24,386 face images, including 21 landmarks and large poses. Of the 21 landmarks, we used only 19 points, excluding the points on the left and right sides of the face. Zhu et al. [49] divided the AFLW dataset into 20,000 images as the training set and 4386 images as the test set, and separately evaluated 1314 images in the test set for a performance evaluation of the frontal face. Figure 9 shows the good results of the CHS for various poses. As shown in Table 3, we applied an evaluation according to each criterion. The CHS showed performance improvements of 8.6% and 2.3% for full and frontal NME*_diag_* in comparison to FaRL [23] and 2.2% for full NME*_box_* in comparison to DTLD [24], respectively. Figure 10 shows the CED curves of the test samples for the CHS, LUVLi, and KDN results. As the figure indicates, the CHS (blue curve) has a higher proportion of samples with fewer errors than LUVLi (orange curve) and KDN (green curve).

#### 4.3.3. Evaluation of COFW

**Figure 11:**
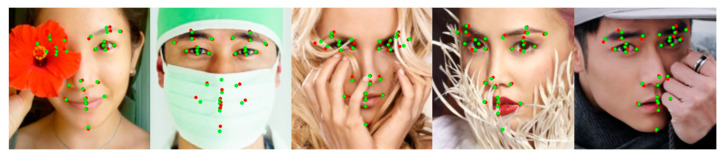
Visualized examples on the COFW datasets. Green dots denote predictions by the CHS, and red dots denote the ground truth.

The COFW consists of 1315 training sets and 507 test sets, including large occlusions, and provides 29 landmarks. Since the size of the COFW training set is smaller than other datasets, the generalization of the network is important for the evaluation. Figure 11 shows the good performance of the CHS on largely occluded samples of the COFW datasets. As shown in Table 4, the CHS obtained NMEs of 4.56 and 3.16 in IPD and IOD metrics, respectively. This result showed improved performance of 2.6% and 0.6% compared to the state-of-the-art IPD and IOD metrics, and a reduced failure rate of 33.9% compared to ADNet [47] in the IPD metric. The low failure rate of the CHS showed the generalization performance and robustness for large occlusions on the COFW.

#### 4.3.4. Evaluation of WFLW

**Figure 12:**
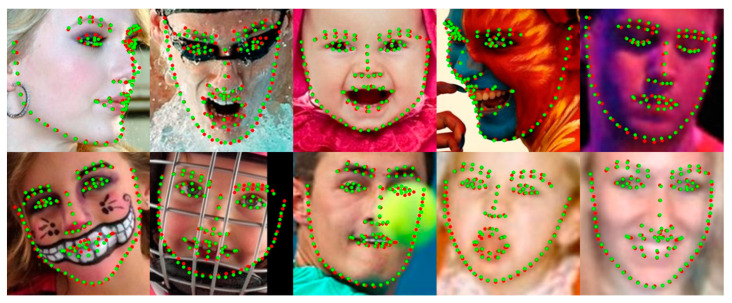
Visualized examples on the WFLW test dataset. Green dots denote predictions by the CHS, and red dots denote the ground truth.

As shown in Table 5, the CHS obtained NMEs of 4.04, 6.76, 4.33, 3.98, 3.87, 4.71, and 4.64 for the entire test set, pose, expression, illumination, make-up, occlusion, and blur subsets, respectively. We improved the performance by 0.5%, 5.3%, 2.9%, 0.5%, −1.6%, 4.8%, and 0.2%, respectively, compared to SPIGA [39]. This result shows a higher performance for the pose and occlusion subsets than other subsets. From the experimental results of the WFLW test set, Figure 12 shows the landmark detection results of CHS for various noises.

## 5. Ablation Study

In this section, we present four ablation studies. The proposed CHS network performs multi-task learning, which trains a heatmap regression task and CCR tasks. We evaluated the contribution of the components to network models that deal with heatmap-CCR networks, loss weights, and selective feature modules. In addition, we compared the performance of the proposed selective feature attention to other attention methods. For effective multi-task learning, we evaluated the performance of the proposed network for backward propagation connections between tasks. Finally, we compared the cost with the networks of other studies through an experiment.

### 5.1. Evaluation of Different Components

To evaluate the effectiveness of the CHS, we trained the network models with different components on the 300W dataset. Table 6 lists the performance of the network models. When fixed loss weights and adaptive loss weights for multi-task learning were not applied, the fixed loss weights were set to 1.0 for training. The performance of four stacked hourglass networks (four HGs) using only the AWing [28] loss did not achieve state-of-the-art NME. However, the models of all the proposed configurations showed good performance, and the effects of each element were experimentally proven. When all the components were applied, the performance was improved by 8.33% for the four HGs.

### 5.2. Comparison of Feature Map Attention Methods

**Figure 13:**
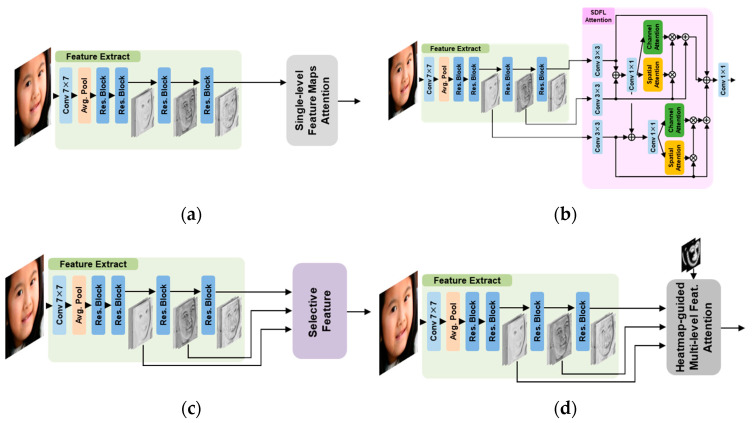
Structures of the feature attention module. We applied these attention modules to our network. (**a**) The single-level feature map attention includes CBAM [42] and Selective Kernel [52]. These use only the feature maps in the last residual block of the feature extractor. (**b**) The attention module in SDFL [21] is designed using the CBAM for the multi-level feature maps. The last 1 × 1 convolution layer after the SDFL attention is for adjusting the feature map dimension. (**c**) The selective feature is proposed by us. (**d**) The heatmap-guided attention is applied to the SDFL and the selective feature. The estimated heatmap concatenates with the feature maps. These are operated before the multi-level feature attention module, such as SDFL attention and selective feature.

We compared the proposed selective feature module with conventional attention modules to evaluate them. Figure 13a shows the attention structure with single-level feature maps, which was proposed by CBAM [42] and a selective kernel [52]. Figure 13b shows the attention structure with multi-level feature maps using the CBAM proposed by SDFL [21]. Figure 13c shows the selective feature module proposed in this study, and Figure 13d shows the structure of heatmap-guided attention with multi-level feature maps. Table 7 shows the evaluation results of the trained model for the 300W dataset by applying different attention modules to the network layer of the four CCRs. Owing to the structural features of the ROI feature maps in the CCR network layer, the performance of the attention modules without the heatmap deteriorated because they used features outside the ROI of the landmark. Meanwhile, heatmap-guided attention improves performance by focusing on features around landmarks. The selective feature module in this study showed 1.45% better performance than the attention of SDFL using multi-level feature maps and heatmaps together.

### 5.3. Evaluation of Different Backward Propagation Settings in the CCR Stage

The CHS is a multi-task learning-based network model consisting of a heatmap regression task and CCR tasks. Each stage of the CCR task generates information propagation between each other because it adds the landmark coordinate in the previous stage task to the coordinate offset in the current stage. In multi-task learning, the connected relationship between each stage of the task significantly affects the learning performance owing to forward or backward propagation. Table 8 shows the results of the trained model for the 300W dataset for the three types of backward propagation connections to the previous stage, as shown in Section 3.2. The CHS proposed in this study showed good performance in the connected CCRs. It shows that backward propagation in the CCR network layer degrades the performance at the end of the heatmap regression network, but the feature extractor shared by the heatmap regression network and CCR network improves not only the performance of heatmap regression but also the performance of backward propagation between CCRs.

### 5.4. Model Complexity

**Figure 14:**
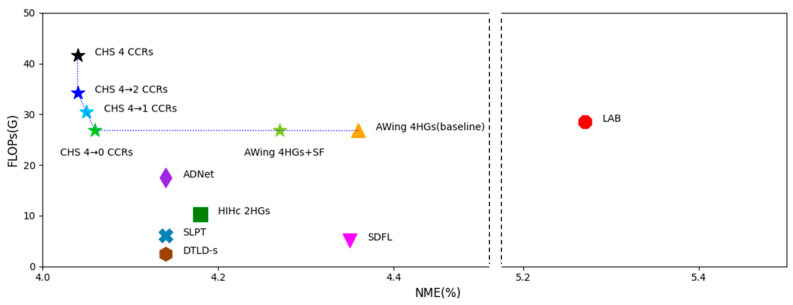
NME of the WFLW test dataset versus FLOPs. Our CHSs outperformed other methods in terms of the NME. In particular, the pruned CHSs maintained their performance while reducing the computational cost.

The CHS requires a relatively high computational load. The proposed heatmap regression network is based on the AWing [28] network, which consists of many parameters, and the CCR network also consists of many parameters due to the independent convolution kernels having each landmark after the ROI pooling. However, as described in Section 3.3, CCR pruning reduces the computational cost while maintaining the performance of the NME. Table 9 and Figure 14 present the results of the network models trained on the WFLW dataset for comparison with other state-of-the-art models. Although the number of parameters of the four CCR networks proposed in this study is large, the NME’s performance is the best. With the proposed one-CCR network, the pruning model of the one-CCR network slightly increased 1.14 times in FLOPs compared to the AWing network but achieved a 7.1% improvement in the NME. A network with all CCRs pruned has the same structure as the AWing network using the selective feature. The computational cost of this network increases very slightly compared to the AWing network, but the network with all CCRs pruned shows better performance than the AWing network with the selective feature trained from scratch. Because the feature extractor and the heatmap regression network are improved by multi-task learning with CCR.

## 6. Discussion

This study improved the quality of feature maps and the performance of face alignment using heatmap-guided selective feature attention and the multi-task learning-based network.

The heatmap-guided selective feature attention selected valid feature maps, which are robust against noisy environments, from multi-level feature maps with different properties. Figure 4 shows the visualized feature maps with reduced noise by the proposed method. In Section 4, the proposed method obtained good results even on noisy datasets, including the 300W challenging subset, AFLW, and COFW. The proposed method also significantly improved 5.3% and 4.7% compared to the previous best one in the WFLW pose and occlusion subsets, respectively.

The multi-task learning-based network controlled the information propagation using the proposed backward propagation connection at the last layer connected between task stages of the CHS. Table 8 shows the performance improvement of the CCR connection, which connects to the same type of task. In addition, the proposed network showed good generalization performance. As shown in Table 5, the CHS performed better on FR_10_ relative to other evaluation metrics. This result means that the proposed method makes fewer estimation failures with challenging data.

Previous face alignment methods based on multi-task learning trained regression tasks separately [9,10] or with other facial properties [37,38]. This study demonstrated the positive effect of multi-task learning by improving the performance of the proposed network trained with the same objective tasks, which are coordinate and heatmap regression for facial landmark detection. As shown in Table 9, the network trained from scratch with selective feature attention and four-stacked hourglass networks but without CCRs obtained a 4.27 NME. However, the network trained with all components and 4-CCRs obtained a result of 4.06 NME, which improved by 4.9% even after pruning the 4-CCRs. 

**Figure 15:**
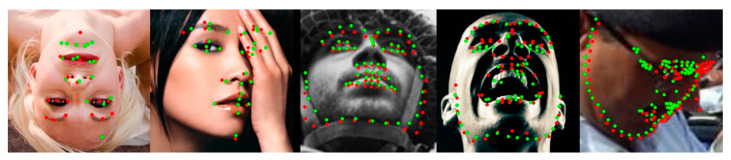
Visualized failure examples on all kinds of datasets. Green dots denote predictions by the CHS, and red dots denote the ground truth.

The proposed method showed robust performance on most face images but bad performance in a few large pose face images. The leading cause of failure in large pose cases is that all the datasets used in learning have many samples with the frontal pose but few with large poses. Similar failure cases are also observed in other face alignment methods. Figure 15 shows several failure cases of the proposed method. However, as shown in Section 4 comparison experiments, the performance of all face alignment methods is low on the challenging subset of 300W and the pose subset of WFLW, which contain relatively large pose face images. However, the proposed method in this paper performs better than other methods in large pose subsets. To deal with the imbalance of samples for large poses, we will improve in future work through oversampling, such as pose-based data balancing [18] or data augmentation using synthesis samples such as GEAN [48].

## 7. Conclusions

We propose a heatmap-guided selective feature attention and a multi-task learning-based network with a refinement strategy that combines a heatmap regression task and cascaded coordinate regression tasks for face alignment. 

The proposed method improves the quality of feature maps by using valid feature maps, which are selected from multi-level feature maps. Multi-level feature maps provide rich information on various properties, and heatmap-guided selective attention helps to select valid feature maps by focusing on facial components. The designed backward propagation connection improves multi-task learning of the proposed network with coordinate and heatmap regression tasks.

Various experiments have shown that the proposed method is superior to conventional methods. The proposed method is 5.3% and 4.7% higher than the previous best one in NME performance for WLFW pose and occlusion subsets, respectively. 

Ablation studies have shown that the proposed method improves the performance of face alignment. Each proposed component has demonstrated effectiveness through evaluations of various network structures in which the components are combined. The heatmap-guided selective feature attention has been compared to other attention methods, and an experiment on designing backward propagation connections finds that the CCR connection with the same task type helps multi-task learning. 

The proposed method has shown robust performance on most face images but has failed on large pose face images because most of the datasets consist of mainly frontal pose face images. In the future, we plan to study methods to improve performance using data balancing.

## Figures and Tables

**Table 1:** Evaluation of the 300W public test set. The best scores are denoted in bold.

Normalization	Method	Common Subset	Challenging Subset	Fullset
Inter-Pupil Distance Normalization	3DDE [35]	3.73	7.10	4.39
	Wing [18]	**3.27**	7.18	**4.04**
	LAB [9]	3.42	6.98	4.12
	AWing [28]	3.77	6.52	4.31
	LRefNet [19]	3.76	6.89	4.37
	SHN-GCN [29]	3.78	6.69	4.35
	SLD [20]	3.64	6.88	4.27
	ADNet [47]	3.51	**6.47**	4.08
	SPIGA [39]	3.59	6.73	4.20
	CHS (Ours)	3.49	**6.47**	4.07
Inter-Ocular Distance Normalization	3DDE [35]	2.69	4.92	3.13
	LAB [9]	2.98	5.19	3.49
	LRefNet [19]	2.71	4.78	3.12
	AWing [28]	2.72	4.52	3.07
	LUVLi [38]	2.76	5.16	3.23
	SHN-GCN [29]	2.73	4.64	3.10
	GEAN [48]	2.68	4.71	3.05
	ACN [10]	2.56	4.81	3.00
	HIH [8]	2.93	5.00	3.33
	SDFL [21]	2.88	4.93	3.28
	SLD [20]	2.62	4.77	3.04
	ADNet [47]	2.53	4.58	2.93
	FaRL (Scratch) [23]	2.90	5.19	3.35
	SLPT [25]	2.75	4.90	3.17
	DTLD-s [24]	2.67	4.56	3.04
	SPIGA [39]	2.59	4.66	2.99
	CHS (Ours)	**2.52**	**4.48**	**2.91**

**Table 2:** Evaluation of the 300W private test set and the COFW-68 dataset. The best scores are denoted in bold.

Method	300W Private	COFW-68
SHN [27]	4.05	-
LAB [9]	-	4.62
3DDE [35]	3.74	-
LRefNet [19]	-	4.40
AWing [28]	3.56	-
GEAN [48]	-	4.24
ACN [10]	3.55	3.83
SLD [20]	-	4.22
SDFL [21]	-	4.18
SLPT [25]	-	4.10
SPIGA [39]	3.43	3.93
CHS (Ours)	**3.35**	**3.78**

**Table 3:** Evaluation of the AFLW dataset. The best scores are denoted in bold.

Bounding Box	Method	NME*_diag_*		NME*_box_*		AUC_7,*box*_
		Full	Frontal	Full	Frontal	Full
Dataset Bounding Box	CCL [49]	-	-	2.27	2.17	-
	LAB [9]	-	-	1.84	1.62	-
	Wing [18]	-	-	1.65	-	-
	SAN [50]	-	-	1.91	1.85	-
	3DDE [35]	-	-	2.01	-	
	SHN-GCN [29]			2.15	-	-
	LRefNet [19]	-	-	1.63	1.46	-
	AWing [28]	-	-	1.53	1.38	-
	GEAN [48]	-	-	1.59	1.34	-
	LUVLi [38]	1.39	1.19	-	-	-
	FaRL (Scratch) [23]	1.05	0.88	1.48		79.3
	DTLD-s [24]	-	-	1.39	-	-
	CHS(Ours)	**0.96**	**0.86**	**1.36**	**1.23**	**81.1**
GT Bounding Box	SAN [50]	-	-	4.04	-	54.0
	Wing [18]	-	-	3.56	-	53.5
	KDN [51]	-	-	2.80	-	60.3
	LUVLi [38]	-	-	2.28	-	68.0
	CHS(Ours)	**1.26**	**1.08**	**1.91**	**1.55**	**73.5**

**Table 4:** Evaluation of the COFW dataset. The best scores are denoted in bold.

Normalization	Method	NME (↓)	FR (↓)	AUC_10_ (↑)
Inter-Pupil Distance Normalization	SHN [27]	5.60	-	-
	Wing [18]	5.44	3.75	-
	3DDE [35]	5.11		
	AWing [28]	4.94	0.99	**0.6440**
	SHN-GCN [29]	5.67	-	**-**
	ADNet [47]	4.68	0.59	0.5317
	SLPT [25]	4.79	1.18	-
	CHS (Ours)	**4.56**	**0.39**	0.5441
Inter-Ocular Distance Normalization	SHN [27]	4.00	-	-
	LAB (wo/B) [9]	5.58	2.76	
	SDFL [21]	3.63	**0.00**	-
	HIH [8]	3.28	**0.00**	0.6720
	DTLD-s [24]	3.18	**-**	-
	SLPT [25]	3.32	**0.00**	-
	CHS (Ours)	**3.16**	**0.00**	**0.6833**

**Table 5:** Evaluation of the WFLW dataset. The best scores are denoted in bold.

Method	Test Set	PoseSubset	Expression Subset	Illumination Subset	Make-Up Subset	Occlusion Subset	BlurSubset
NME (↓)							
LAB [9]	5.27	10.24	5.51	5.23	5.15	6.79	6.32
3DDE [35]	4.68	8.62	5.21	4.65	4.60	5.77	5.41
AWing [28]	4.36	7.38	4.58	4.32	4.27	5.19	5.32
LUVLi [38]	4.37	7.56	4.77	4.30	4.33	5.29	4.94
AnchorFace [22]	4.32	7.51	4.69	4.20	4.11	4.98	4.82
HIH [8]	4.18	7.20	**4.19**	4.45	3.97	5.00	4.81
SDFL [21]	4.35	7.42	4.63	4.29	4.22	5.19	5.08
SLD [20]	4.21	7.36	4.49	4.12	4.05	4.98	4.82
ADNet [47]	4.14	6.96	4.38	4.09	4.05	5.06	4.79
FaRL (Scratch) [23]	4.80	8.78	5.09	4.74	4.99	6.01	5.35
DTLD-s [24]	4.14	-	-	-	-	-	-
SLPT [25]	4.14	6.96	4.45	4.05	4.00	5.06	4.79
SPIGA [39]	4.06	7.14	4.46	4.00	**3.81**	4.95	4.65
CHS (Ours)	**4.04**	**6.76**	4.33	**3.98**	3.87	**4.71**	**4.64**
FR_10_ (↓)							
LAB [9]	7.56	28.83	6.37	6.73	7.77	13.72	10.74
3DDE [35]	5.04	22.39	5.41	3.86	6.79	9.37	6.72
AWing [28]	2.84	13.50	2.23	2.58	2.91	5.98	3.75
LUVLi [38]	3.12	15.95	3.18	2.15	3.40	6.39	3.23
AnchorFace [22]	2.96	16.56	2.55	2.15	2.43	5.30	3.23
HIH [8]	2.96	15.03	**1.59**	2.58	**1.46**	6.11	3.49
SDFL [21]	2.72	12.88	**1.59**	2.58	2.43	5.71	3.62
SLD [20]	3.04	15.95	2.86	2.72	1.45	5.29	4.01
ADNet [47]	2.72	12.72	2.15	2.44	1.94	5.79	3.54
FaRL (Scratch) [23]	5.72	-	-	-	-	-	-
DTLD-s [24]	3.44	-	-	-	-	-	-
SLPT [25]	2.76	12.27	2.23	1.86	3.40	5.98	3.88
SPIGA [39]	2.08	11.66	2.23	**1.58**	**1.46**	4.48	**2.20**
CHS (Ours)	**1.80**	**9.51**	**1.59**	1.72	**1.46**	**3.13**	2.46
AUC_10_ (↑)							
LAB [9]	0.5323	0.2345	0.4951	0.5433	0.5394	0.4490	0.4630
3DDE [35]	0.5544	0.2640	0.5175	0.5602	0.5536	0.4692	0.4957
AWing [28]	0.5719	0.3120	0.5149	0.5777	0.5715	0.5022	0.5120
LUVLi [38]	0.5770	0.3100	0.5490	0.5840	0.5880	0.5050	0.5250
AnchorFace [22]	0.5769	0.2923	0.5440	0.5865	0.5914	0.5193	0.5286
HIH [8]	0.5970	0.3420	**0.5900**	0.6060	0.6040	0.5270	0.5490
SDFL [21]	0.5759	0.3152	0.5501	0.5847	0.5831	0.5035	0.5147
SLD [20]	0.5893	0.3150	0.5663	0.5953	0.6038	0.5235	0.5329
ADNet [47]	0.6022	0.3441	0.5234	0.5805	0.6007	0.5295	0.5480
FaRL (Scratch) [23]	0.5454	-	-	-	-	-	-
SLPT [25]	0.5950	0.3480	0.5740	0.6010	0.6050	0.5150	0.5350
SPIGA [39]	**0.6056**	0.3531	0.5797	**0.6131**	**0.6224**	0.5331	**0.5531**
CHS (Ours)	0.6015	**0.3552**	0.5792	0.6080	0.6155	**0.5403**	0.5462

**Table 6:** Evaluation of different components. The best scores are denoted in bold.

Component	Choice										
4-HGs	✓	✓	✓	✓	✓	✓	✓	✓	✓	✓	✓
Selective Feature	-	✓	-	-	-	-	✓	✓	✓	✓	✓
2-CCRs									✓		-
4-CCRs	-	-	✓	✓	✓	✓	✓	✓	-	✓	-
6-CCRs									-		✓
Adaptive Weight	-	-	-	✓	-	✓	✓	-	✓	✓	✓
Fixed Loss Weight	-	-	-	-	✓	✓	-	✓	✓	✓	✓
NME (↓)	4.44	4.24	4.20	4.18	4.15	4.14	4.12	4.11	4.12	**4.07**	4.10

**Table 7:** Evaluation of different attention modules. The best scores are denoted in bold.

Evaluation	Without Attention	Single-Level, without Heatmap		Multi-Level			
				without Heatmap		Heatmap-Guided	
		CBAM [42]	SK [52]	SDFL [21]	SF (Ours)	SDFL [21]	SF (Ours)
NME (%)	4.14	4.17	4.17	4.17	4.15	4.13	**4.07**

**Table 8:** Evaluation of different backward propagation settings. NME_heatmap-stage_ and NME_4th-CCR-stage_ are checked values in each task stage. NME_heatmap-stage_–NME_4th-CCR-stage_ is the difference of both NMEs. The best scores are denoted in bold.

Method	NME_heatmap-stage_ (%)	NME_4th-CCR-stage_ (%)	NME_heatmap-stage_–NME_4th-CCR-stage_
Task-Wise Connection	4.22	4.11	0.11
Fully Connection	4.24	4.11	0.13
CCRs Connection	**4.21**	**4.07**	**0.14**

**Table 9:** Model complexity.

Method	#Params (M)	FLOPs (G)	NME (↓)
LAB [9]	32.05	28.58	5.27
AWing (baseline) [28]	24.15	26.79	4.36
SDFL [21]	24.68	5.17	4.35
HIHc (2 HGs) [10]	14.47	10.29	4.18
ADNet [47]	13.48	17.47	4.14
DTLD-s [24]	13.30	2.50	4.14
SLPT [25]	13.19	6.12	4.14
AWing 4HGs + SF	24.15	26.80	4.27
CHS 4 CCRs	154.04	41.69	4.04
CHS 4 → 2 CCRs	89.09	34.25	4.04
CHS 4 → 1 CCR	56.62	30.52	4.05
CHS 4 → 0 CCR	24.15	26.80	4.06

## Data Availability

Not applicable.
